# Association of Eating Pattern, Chronotype, and Social Jetlag: A Cross-Sectional Study Using Data Accumulated in a Japanese Food-Logging Mobile Health Application

**DOI:** 10.3390/nu15092165

**Published:** 2023-04-30

**Authors:** Lyie Nitta, Yu Tahara, Takae Shinto, Saneyuki Makino, Mai Kuwahara, Ayako Tada, Nanako Abe, Mikiko Michie, Shigenobu Shibata

**Affiliations:** 1 Laboratory of Physiology and Pharmacology, School of Advanced Science and Engineering, Waseda University, Tokyo 162-0056, Japan; lyn@ruri.waseda.jp (L.N.); y53-m-h423@moegi.waseda.jp (T.S.); s.makino@fuji.waseda.jp (S.M.); kmykmya@akane.waseda.jp (M.K.); shibatas@waseda.jp (S.S.); 2 Graduate School of Biomedical and Health Sciences, Hiroshima University, Hiroshima 734-0037, Japan; 3 Asken Inc., Tokyo 163-1408, Japan; tadaay@greenhouse.co.jp (A.T.); abena@greenhouse.co.jp (N.A.); mikiko-michie@greenhouse.co.jp (M.M.)

**Keywords:** circadian clock, sleep, night owl, phone app, micronutrients, chronotype, chrono-nutrition, social jetlag

## Abstract

Chronotype (morningness–eveningness) and social jetlag (SJL; discrepancy in the sleep pattern between the weekday and weekend) are related to eating behavior and health. The association between sleep behavior and the daily macro- and micronutrient eating pattern of each meal (breakfast, lunch, and dinner) have not been discussed well and need more evidence. Here, meal pattern datasets of Japanese participants aged 20–59 years were obtained as averages over 1 month from the data stored in the food-logging app “Asken”. We allocated three groups for each chronotype and SJL. Multiple regression analyses revealed that morning chronotype and small SJL were associated with higher total daily intake of potassium, fiber, magnesium, phosphorus, and vitamin K. Breakfast energy intake and consumption of nutrients, including protein, lipid, carbohydrate, and minerals, were higher in the morning chronotype or small SJL. Lunch intake of potassium, cholesterol, fiber, magnesium, and vitamin K was also higher in the morning chronotype or small SJL. Dinner energy intake and nutrient intake of proteins, lipids, carbohydrates, sodium, and saturated fatty acids were lower in the morning chronotype or small SJL. The current data would help to establish a detailed reference for dietary intake which considers eating patterns over a day.

## 1. Introduction

Chronotype (morningness–eveningness) describes different timings of comfortable living for individuals, and is determined by genetic, environmental, and social factors [1]. Those with the evening type tend to have a longer circadian clock period than those with the morning type do, and their daily rhythms are more easily delayed [2]. Genome-wide association studies revealed 351 single-nucleotide polymorphisms associated with chronotypes [3]. Evening individuals are forced to lead a morning lifestyle on workdays, owing to social restrictions, resulting in a discrepancy between their workdays and free days lifestyles. This weekly jetlag-induced problem is called “social jetlag” (SJL) [4]. Evening chronotypes and SJL are associated with obesity, lower academic achievement, depression, lower performance, and increased smoking habits [4,5,6]. Interventions for chronotypes and SJL prevent lifestyle-related diseases [7]; however, only a few intervention trials have been reported. The COVID-19 pandemic has delayed workday wake-up times and reduced SJL by prompting quarantining and working from home [8,9]. Although most Japanese participants in a study had increased body weight and decreased daily physical activity, associations with advances in sleep timing and weight loss and delays in sleep timing and weight gain were observed during the pandemic-related home quarantine in 2020 [8]. Improvements in sleep and daily performance have also been reported with a change to later school start times [10]. Thus, the individual characteristics of the circadian clock need to be carefully considered, and further cross-sectional and interventional research evidence on chronotype/SJL needs to be accumulated.

Evening chronotypes or people with SJL have higher breakfast skipping rates, and they consume more evening meals [11,12,13]. They also have irregular mealtimes, which are associated with obesity. Although eating patterns and nutritional contents related to chronotype have been reported, and studies have discussed the total daily amounts well, only a few studies have investigated meal contents [11,12,13]. According to a recent systematic review, eight studies indicated no difference in total daily macronutrient intake among chronotypes, and three studies revealed inconsistent differences in daily carbohydrate intake among chronotypes [14]. In addition, three studies have reported that morning chronotypes have higher macronutrient intake in the morning, and evening chronotypes have higher macronutrient intake in the evening [14]. Mito et al. reported that evening chronotype female students had a lower total daily intake of protein, calcium, magnesium, zinc, vitamins (D, riboflavin, and B6), and vegetables, and consumed more noodles [15]. In older women, the evening chronotype consumed less vitamin D, more bread, and more caffeinated beverages [16]. Yoshizaki et al. reported that higher SJL is related to a lower total daily energy intake, lower grain consumption, and higher sugar and confectionery consumption [17]. A previous study in Brazil revealed that the higher SJL group consumed more calories, saturated fat, and cholesterol during dinner; more protein, total fat, saturated fat, and cholesterol during lunch; and more total fat and saturated fat in morning snacks [18]. However, daily micronutrient eating patterns related to chronotype or SJL need to be investigated in a large sample size with a broad age range [14]. Therefore, this study aimed to examine the differences in eating patterns among chronotypes and SJL based on breakfast, lunch, and dinner meal data obtained from users of a Japanese food-logging mobile health application (mHealth app).

## 2. Materials and Methods

### 2.1. Ethical Considerations

This study was approved by the Ethics Review Committee on Research with Human Subjects at Waseda University (No. 2020-046), and was conducted in accordance with the guidelines of the Declaration of Helsinki. A cross-sectional study was designed, conducted, and analyzed according to the STROBE statement [19]. Informed consent was obtained from all individuals who participated in the study when they started using the app and answered the web survey. The participants completed the questionnaire anonymously to protect their privacy and maintain confidentiality.

### 2.2. Food-Logging mHealth App “Asken”

“Asken” is a popular Japanese food-logging and food-coaching mHealth app, downloaded by approximately over 7,329,000 users in May 2022 [20]. Self-reported food logs accumulated in the app have been deemed reliable for research purposes [21,22]. As most users (almost 95%) used this app for body weight reduction (evidence from another of our surveys for the same app users), and women may be more inclined to maintain their body shape than men, about 70% of the app users were women (evidence from the current data). Users can input ingredients, dishes and their portion sizes into the app. The app automatically calculates calorie intake and nutritional intake from food records by referencing the Standard Tables of Food Composition in Japan (Ministry of Education 2015). Detailed calculations of nutrient amounts using the food-logging mHealth app have been previously described [21,22]. The app also provides feedback on the value of nutrition intake based on the Dietary Intake Standards for the Japanese, as determined by the Ministry of Health, Labor, and Welfare [23].

### 2.3. Participants, and Data Inclusion and Exclusion

An online survey was conducted among Asken users at the end of January 2021. Amazon gift cards (500 Japanese yen) were offered to the participants in the lottery. We only set the age range (20–59 years), leaving out any disease information for inclusion and exclusion criteria in the online survey (6299 app users). We selected participants who recorded food logs for 10 or more days per month (848 people were excluded), reported their gender, and were non-shift workers (825 people were excluded). Finally, 4626 participants were included (3427 women and 1199 men, Table 1).

### 2.4. Dietary Data

The average dietary data during the 1-month period (January–February 2021) were used for the analysis. The current study used data on the energy and intake of 26 nutrients (protein, fat, carbohydrates, sodium, potassium, cholesterol, dietary fiber, saturated fatty acids, alcohol, calcium, magnesium, phosphorus, iron, zinc, and vitamins A, B1, B2, B3, B5, B6, B12, C, D, E, K, and folate) at breakfast, lunch, and dinner. Missing values in food logs were not included in the average calculation because we could not verify whether they were due to meal skipping or data omission. Although the food log includes a snack category, we did not use this data in the current analysis because of insufficient information on intake time. Because 99.7% of the data were included within 3× standard deviations of the distribution, the other 0.3% of the values were excluded as outliers in each category. The app also collected data on daily body weight, body fat percentage, step count, and time of food intake, if the users input the data or connected other health apps to the Asken. 

### 2.5. Questionnaires

We asked participants to complete 50 questions, including 7 items on basic characteristics (age, self-reported gender, weight, height, prefecture of residence, shift work status, and regularity of lifestyle), 6 items on eating behavior, and 6 items on physical activity (a short version of the International Physical Activity Questionnaire) [24], subjective well-being, health, and physical fitness. For sleep behavior, we used the short version of the Munich Chrono Type Questionnaire to investigate sleep habits and chronotypes using 10 questions [25]. Some 8 items of the Athene Insomnia Scale (AIS) were used to assess sleep problems [26]. Breakfast or late-night snack frequency (days/week), subjective well-being, health, and physical fitness were assessed using a 5-step selection questionnaire: 1 = strongly disagree, 2 = disagree, 3 = neither agree nor disagree, 4 = agree, and 5 = strongly agree [27].

### 2.6. Grouping of Chronotype and SJL

To group chronotypes, mid-sleep on free days corrected for sleep debt accumulated during workdays (MSFsc) was used [1]. We divided the users into three groups based on MSFsc (morning: 3 < MSFsc; intermediate: 3 ≤ MSFsc < 5; evening: 5 ≤ MSFsc). SJL was calculated as the difference between the midpoints of sleep on workdays and free days. SJL was classified into three groups: small (SJL < 1 h), medium (1 h ≤ SJL < 2 h), and large SJL (SJL ≤ 3 h). 

### 2.7. Statistical Analyses

Based on our previous findings from cross-sectional studies using food-logging app data [28], a power analysis was applied before the current experiment (G*Power, version 3.1.9, Heinrich-Heine-University, Düsseldorf, Germany). The analysis was conducted using SPSS software (version 27.0; IBM Corp., Armonk, NY, USA) (Faul et al. 2007). Data normality was analyzed using a Kolmogorov–Smirnov test. As most samples did not pass the normality test, a non-parametric analysis was used in this study. A Kruskal–Wallis test was conducted for multiple-sample statistics to understand the chronotype- or SJL-related general characteristics and eating behavior in women and men (Table 2, Table 3, Table 4 and Table 5). *p* < 0.001 by Kruskal–Wallis test with a coefficient of more than 0.2, or lower than −0.2 by Spearman’s correlation was considered a significant difference among groups. A multiple regression analysis (forced entry method) was conducted to determine the association between chronotype (1: morning, 2: intermediate, 3: evening) and SJL (1: small SJL, 2: medium SJL, 3: large SJL) and each nutriment intake in each meal timing, with confounding factors (age, gender, BMI, and total daily intake) (Table 6, Table 7, Table 8 and Table 9). Statistical significance was set at *p* < 0.001.

## 3. Results

### 3.1. Basic Characteristics

The general characteristics of the study group are indicated in Table 1. As described previously [28,29], the ratio of carbohydrate intake within the macronutrients in the current participants was lower than the Dietary Intake Standards for the Japanese, which is 50–65% carbohydrate in all ages. Interestingly, in a day, the carbohydrate ratio was higher at breakfast and lower at dinner. 

Chronotype-dependent analyses are shown in Table 2 for women and Table 3 for men. Compared with morning types, evening types had a younger age; a later eating time for breakfast, lunch, and dinner; a more irregular lifestyle; larger SJL; and more frequent breakfast skipping in both women and men (Table 2 and Table 3; *p* < 0.001 by a Kruskal–Wallis test, with coefficient of more than 0.2 or lower than −0.2, Spearman’s correlation). In women, evening types also showed a later eating time for lunch and dinner. Based on only the Kruskal–Wallis test, evening types demonstrated higher body weight, smaller breakfast intake, bigger dinner, a larger standard deviation (SD) of eating time (suggesting irregularity in their daily eating times), more frequent night snacks, more sleep problems (measured by the AIS questionnaire) and lower scores for subjective health and well-being in women (Table 2). 

As presented in Table 4 and Table 5, larger SJL demonstrated a later MSFsc in both genders. Based on only the Kruskal–Wallis test, larger SJL correlated with younger age, higher daily intake, bigger dinner, a later dinner time, larger SD of breakfast time, irregular lifestyle, more frequent breakfast-skipping, a higher AIS score, and lower subjective physical strength, health, and well-being in women (Table 4). Similar differences were seen in men (Table 5).

### 3.2. Chronotype/SJL Associated Eating Pattern

Multiple regression analyses were conducted to understand the association between eating patterns and chronotypes or SJL. Age, gender, body mass index (BMI), and total daily intake (kcal) were adjusted as confounding variables. For the total daily intake of each nutrient (Table 6), potassium, fiber, magnesium, phosphorus, and vitamin K were negatively associated with chronotype and SJL, suggesting that morning types or people with small SJL had more potassium, fiber, magnesium, phosphorus, and vitamin K in a day. The trend of each nutrient intake basically showed higher intake at breakfast and lower intake at dinner in the morning-type and small SJL group, compared with the evening-type and large SJL group (Tables S1 and S2). 

At breakfast (Table 7), energy intake and many nutrients including proteins, lipids, carbohydrates, sodium, potassium, cholesterol, fiber, saturated fatty acid, calcium, magnesium, phosphorus, zinc, vitamin K, folate, and pantothenic acid were negatively associated with chronotype. Similar results were observed between breakfast eating patterns and SJL (Table 7). These results suggest that the morning chronotypes or those with small SJL eat more food during breakfast. 

At lunch (Table 8), potassium, cholesterol, fiber, magnesium, and vitamin K levels demonstrated a common negative association with chronotype/SJL. 

In contrast with breakfast intake, the dinner energy intake of each nutrient, including proteins, lipids, carbohydrates, sodium, and saturated fatty acids, were positively associated with chronotype or SJL (Table 9).

## 4. Discussion

In the present study, data from 1-month dietary records and web-based surveys were analyzed across a broad age distribution, and differences in eating patterns among chronotypes and SJL based on breakfast, lunch, and dinner meal were investigated. We obtained all the previously reported basic characteristics associated with chronotype/SJL [11,12,13]. We identified mealtimes, irregular mealtimes, breakfast/late-night snack intake frequency, exercise habits, and sleep problems related to chronotype and SJL. Systematic reviews revealed an association between chronotype and nutrient intake; however, the data were research-dependent, because of the research-specific differences in country, age, gender, and chronotype determination methodology [11,12,13,30,31]. In addition, the micronutrient eating pattern related to chronotype or SJL has not been investigated [14]. Here, we newly captured the daily eating patterns of 26 macro- and micronutrients at breakfast, lunch, and dinner against chronotype and SJL in a large sample size with a broad age range.

We identified lower total daily intakes of potassium, fiber, magnesium, phosphorus, and vitamin K in the evening types and large SJL group. Potassium controls fluid balance and blood pressure [32]. Dietary fiber intake is beneficial for the microbiome and good for immune function and cardiovascular function [33]. Magnesium, a cofactor in many enzymes for the cell function, is also beneficial for the muscle function, blood glucose control, and blood pressure regulation [34]. Phosphorus is a component of the cell membrane, nucleic acids, and ATP. Although higher intake of phosphorus has a negative impact on chronic kidney disease and cardiovascular disease, phosphorus is also an important essential mineral [35]. Vitamin K plays an important role in coagulation and bone metabolism [36]. Thus, compared with the evening-type and large SJL group, the morning-type and small SJL group showed a healthier dietary condition.

Some of the nutrients listed above were newly detected when compared with previous studies [14]. Sato-Mito et al. reported that among female university students, night owls had a lower intake of protein, calcium, magnesium, zinc, vitamins (D, riboflavin, and B6), and vegetables, and consumed more noodles [15]; their magnesium levels were consistent with our results. A higher SJL is associated with lower adherence to Mediterranean food, fruits, vegetables, and fiber, and a higher intake of fast food, sugar, confectionaries, and sweetened drinks [14,17,37,38,39]. Thus, since fruits and vegetables are rich in minerals and fiber, we can deduce that lower potassium, fiber, and magnesium intake might be a common feature of people with higher SJL.

The current study revealed the nutrient eating patterns of different chronotypes and levels of SJL. Breakfast energy intake and nutrient intake of proteins, lipids, carbohydrates, and minerals were positively associated with morning chronotype or small SJL, suggesting a bigger breakfast intake in people with the morning chronotype or small SJL. Lunch intake of potassium, cholesterol, fiber, magnesium, and vitamin K was also positively associated with the morning chronotype or small SJL. Dinner energy intake and nutrient intake of proteins, lipids, carbohydrates, sodium, and saturated fatty acids were negatively associated with the morning chronotype or small SJL. One study addressed eating patterns during each meal of the day, and reported that the large SJL group had more saturated fat and cholesterol intake at dinner and lunch among patients with obesity [18], which is consistent with the current data. A larger breakfast and smaller dinner were common eating behaviors related to the morning type and small SJL, a finding consistent with previous research [11,12,13]. Although causality could not be determined by the current and previous cross-sectional studies, the association between eating habits and chronotype can be explained by the food-entrained circadian clock, based on animal research [40]. Based on the phase–response curve of food stimulation of the circadian clock [41], breakfast consumption accelerates the morningness of the circadian clock, whereas dinner and evening meals promote a nocturnal shift in the internal clock. Foods/nutrients regulate the circadian clock in peripheral tissues through postprandial insulin secretion and insulin-induced acute changes in clock gene expression [42,43]. Therefore, higher insulin secretion owing to morning carbohydrate intake causes a shift in morningness. Magnesium and vitamin K, of which consumption is higher during breakfast in the morning-type and small SJL group, can also help insulin secretion/response and breakfast-induced phase advancement [44,45]. By contrast, higher carbohydrate intake at dinner or late at night may delay the sleep–wake cycle. In fact, dinner carbohydrates demonstrated stronger associations with MSFsc and SJL in the multiple regression analysis. Postprandial glucose at dinner can be inhibited directly by fiber intake at dinner, or by the second-meal effect of fiber intake at lunch or snack time [46,47]. This study identified an association between lunch fibers and morningness or small SJL, which may help reduce dinner-induced phase delay. In addition, fiber intake at breakfast enhances microbiota fermentation and increases short-chain fatty acid production, which accelerates food-induced circadian clock resetting in peripheral tissues [48]. Proteins have also been reported to be entrainment signals of clock gene expression through the IGF-1 pathway [49]. In the current study, dinner protein was related to eveningness, which might be due to protein-induced enhancement in the dinner-induced phase delay. In other words, morning protein promoted morningness. Recently, breakfast protein has been recognized as an important eating habit for maintaining muscle size in both human and mouse studies [50]. Daily physical activity and breakfast protein intake were higher in the morning chronotypes than in the evening chronotypes. Altogether, increasing morning protein levels, but not evening protein levels, is a good health intervention to prevent sarcopenia.

In this study, body weight was significantly different among chronotypes, but not SJL levels. Multiple regression analyses revealed a significant association between BMI and chronotype, but not SJL levels, adjusting for the confounding factors of age and gender (Table S3). However, the association between chronotype and SJL and body weight varies, with conflicting results shown in the literature [11]. SJL is associated with metabolic syndrome in Japanese workers [51]. However, the Chinese population did not indicate any association between BMI and SJL because of their daily napping culture and small SJL [52]. By contrast, Chinese adolescents with higher SJL (>2 h) had a higher BMI [53]. Another study suggested that shorter sleep duration was associated with higher carbohydrate and overall energy intake, and that SJL may be independently associated with increased appetite [54]. In addition, a study on SJL and eating styles in US adolescents predicted a tendency toward increased emotional eating and loss of control over food when SJL was greater [55]. Although no significant difference in the amount of food consumed per day was observed among the groups, the factors associated with the evening type with a higher body weight included a low breakfast and high dinner intake, low fiber throughout the day, a late dinner time, a higher nighttime snack frequency, and irregular mealtimes. These factors have been reported to be causal factors for increased weight [56]. Consuming large amounts of food late at night increases body weight [56]. Irregular eating times are associated with negative mood; “eating jetlag” (the difference in eating time between workdays and free days) was also associated with higher BMI and cardiovascular events [27,57,58]. Glucose tolerance is lower in the evening, and postprandial hyperglycemia is more likely to occur at dinner than at breakfast [59], which might be why the evening type is associated with higher body weight. Thus, evidence for an association between body weight and chronotype-related eating behavior is sufficient.

The limitations of our study included misclassification due to self-reporting, unmeasured and uncontrolled confounding factors, and unclear temporal ordering between variables. Dietary data were self-reported, and there is a possibility that errors and self-efficacy may have occurred. Although the current data included highly accurate food intake data collected across 1 month from a large number of samples of varying ages, research bias was present because 95% of the app users desired weight loss, and 70% of the app users were women. Nutrient data for snacks were not used in this study because the time information of snack intake was missing from the application. A more objective methodology, including actigraphy recordings and observation methods such as sleep diaries, is preferable. The cross-sectional study design limits the determination of causal links between all variables. The current study was conducted in winter, and seasonal effects should be considered [60]. Economic status, marital status, and occupation may also be confounding factors affecting the study, and future research should consider such attributes alongside the confounding factor of social background.

## 5. Conclusions

The current study revealed that people who were morning chronotypes or had small SJL had specific dietary conditions (higher potassium, fiber, magnesium, phosphorus, and vitamin K, with a bigger breakfast and smaller dinner amount) with regular eating and sleeping rhythms. This study provides information for future intervention studies addressing chronotype and SJL, and provides evidence-based personalized dietary suggestions. Since the Dietary Intake Standards for Japanese people do not include the reference intake for each meal (only including a daily total amount), the current data may help to establish a detailed reference intake that considers eating patterns across a day.

## Figures and Tables

**Table 1:** General characteristics of the study group.

		Women		Men	
		N = 3427		N = 1199	
		Mean	SD	Mean	SD
Age (years old)		40.9	10.5	45.6	9.3
Height (cm)		158.7	5.6	171.4	5.9
BMI (kg/m^2^)		21.0	7.3	22.9	7.8
Weight (kg)		58.1	11.0	74.1	13.7
Daily total energy intake (kcal)		1652.9	283.1	2068.9	408.0
Breakfast energy intake (kcal)		349.3	124.7	429.1	154.1
Lunch energy intake (kcal)		518.6	119.8	647.2	145.5
Dinner energy intake (kcal)		572.3	163.8	802.3	200.3
Snacks energy (kcal)		222.8	98.1	263.3	135.4
Daily total	Protein (%)	17.2	4.0	17.2	5.9
	Lipid (%)	32.1	6.2	32.2	9.1
	Carbohydrate (%)	48.8	7.0	49.1	16.4
Breakfast	Protein (%)	17.5	6.9	17.6	8.1
	Lipid (%)	28.2	8.3	27.5	8.8
	Carbohydrate (%)	54.8	11.0	53.0	12.5
Lunch	Protein (%)	16.9	4.3	16.1	4.7
	Lipid (%)	30.8	6.1	30.2	6.1
	Carbohydrate (%)	50.8	7.7	50.4	7.9
Dinner	Protein (%)	19.4	5.1	17.8	5.5
	Lipid (%)	33.7	6.6	32.3	7.0
	Carbohydrate (%)	42.6	9.3	40.6	10.2

% protein, lipids, and carbohydrates were calculated based on the total energy intake in each meal, or total daily intake (kcal).

**Table 2:** Basic characteristics of each chronotype among women.

	Morning		Intermediate		Evening			
	(MSFsc < 3) N = 950		(3 ≤ MSFsc < 5) N = 1890		(5 ≤ MSFsc) N = 587		Kruskal–Wallis Test	Spearman’s
	Mean	SD	Mean	SD	Mean	SD	*p*	Correlation Coefficient
Age (year)	44.0	9.5	40.8	10.5	36.1	10.2	**<0.001**	**−0.24**
Height (cm)	158.5	5.5	158.8	5.6	158.7	5.6	0.658	0.01
BMI	20.7	7.2	21.0	7.2	21.6	7.6	0.002	0.06
Weight (kg)	57.2	11.0	58.1	10.8	59.6	11.8	**<0.001**	0.07
Daily intake (kcal)	1645.3	276.3	1647.6	277.8	1682.0	308.5	0.101	0.02
Breakfast intake (kcal)	379.8	122.8	345.2	119.2	313.3	133.7	**<0.001**	−0.18
Lunch intake (kcal)	517.6	116.9	519.8	116.5	516.7	134.4	0.807	0.00
Dinner intake (kcal)	547.3	165.5	570.7	159.3	617.9	166.2	**<0.001**	0.13
Breakfast time (hh:mm)	7:17	1:16	7:51	1:11	8:52	1:52	**<0.001**	**0.37**
Lunch time (hh:mm)	12:17	1:09	12:29	1:13	13:03	1:35	**<0.001**	**0.20**
Dinner time (hh:mm)	18:44	1:32	19:07	1:18	19:25	2:22	**<0.001**	**0.24**
SD of breakfast time (min)	16.0	31.3	24.2	35.8	34.9	48.5	**<0.001**	0.14
SD of lunch time (min)	12.7	27.7	18.5	31.2	30.2	41.4	**<0.001**	0.16
SD of dinner time (min)	12.5	27.8	18.5	40.4	27.8	55.9	**<0.001**	0.11
Regularities in the rhythm of life (score)	4.1	0.8	3.8	0.9	3.0	1.2	**<0.001**	**−0.35**
MSFsc (h)	2.3	0.6	3.9	0.6	5.7	0.5	**<0.001**	**0.90**
SJL (h)	0.6	0.5	0.9	0.7	1.1	0.8	**<0.001**	**0.24**
Frequency of breakfast (days/week)	6.6	1.4	6.2	1.7	4.8	2.5	**<0.001**	**−0.32**
Frequency of late-night snack (days/week)	1.8	2.7	1.9	2.6	2.1	2.6	**<0.001**	0.08
METs (total physical activity)	34.1	44.4	27.5	34.7	27.5	40.5	0.008	−0.05
AIS score	4.1	3.3	4.4	3.3	5.0	3.6	**<0.001**	0.09
Physical strength (score)	2.8	1.0	2.8	1.0	2.7	0.9	0.015	−0.05
Health (score)	3.7	0.9	3.7	0.9	3.4	0.9	**<0.001**	−0.10
Well-being (score)	3.6	0.9	3.5	1.0	3.4	0.9	**<0.001**	−0.10

SD, standard deviation; BMI, body mass index; SJL, social jetlag; METs, metabolic equivalents; AIS, Athens Insomnia Scale; MSFsc, sleep-corrected midpoint of sleep on free days. *p*-values were calculated using the Kruskal–Wallis test. *p* < 0.001 was considered significant and is presented in bold font. A Spearman’s correlation coefficient of more than 0.2 or lower than −0.2 is presented in bold.

**Table 3:** Basic characteristics of each chronotype among men.

	Morning		Intermediate		Evening			
	(MSFsc < 3)		(3 ≤ MSFsc ≤ 5)		(5 < MSFsc)			
	N = 475		N = 588		N = 136		Kruskal–Wallis Test	Speaman’s
	Mean	SD	Mean	SD	Mean	SD	*p*	Correlation Coefficient
Age (year old)	48.0	8.2	44.7	9.5	40.9	9.9	**<0.001**	**−0.23**
Height (cm)	171.2	5.9	171.6	5.9	171.7	6.0	0.455	0.04
BMI	22.5	7.5	23.0	7.8	24.1	8.2	0.054	0.07
Weight (kg)	72.8	12.7	74.4	13.3	77.3	17.6	0.027	0.08
Daily intake (kcal)	2061.1	415.3	2084.5	402.2	2028.9	406.6	0.383	−0.01
Breakfast intake (kcal)	459.2	149.2	418.4	147.2	371.6	177.0	**<0.001**	−0.18
Lunch intake (kcal)	632.1	145.9	661.6	144.1	637.1	145.1	0.005	0.05
Dinner intake (kcal)	794.9	198.3	808.0	200.9	803.4	205.1	0.586	0.03
Breakfast time (hh:mm)	7:06	1:07	7:40	1:06	8:26	2:04	**<0.001**	**0.34**
Lunch time (hh:mm)	12:16	0:56	12:26	0:55	12:51	1:17	**<0.001**	0.18
Dinner time (hh:mm)	19:13	1:22	19:30	1:14	19:27	2:17	**<0.001**	0.14
SD of breakfast time (min)	25.7	41.8	28.0	39.2	38.6	56.6	0.100	0.06
SD of lunch time (min)	16.2	33.9	20.3	32.0	29.4	45.7	0.004	0.10
SD of dinner time (min)	23.3	46.6	28.9	56.6	46.2	107.1	0.116	0.06
Regularities in the rhythm of life (score)	4.1	0.8	3.9	0.8	3.2	1.2	**<0.001**	**−0.22**
MSFsc (h)	2.1	0.6	3.8	0.5	5.7	0.5	**<0.001**	**0.91**
SJL (h)	0.5	0.5	0.8	0.6	1.2	0.9	**<0.001**	**0.32**
Frequency of breakfast (days/week)	6.4	1.6	6.2	1.8	5.3	2.4	**<0.001**	**−0.20**
Frequency of late night snack (days/week)	2.0	2.9	2.2	2.8	2.9	2.9	**<0.001**	0.11
Mets (total physical activity)	39.9	43.4	35.8	36.8	30.6	33.2	0.008	−0.09
AIS score	3.9	3.3	4.1	3.2	4.5	3.3	0.047	0.07
Physical strength (score)	3.1	1.0	3.0	0.9	3.0	1.0	0.328	−0.04
Health (score)	3.9	0.8	3.7	0.9	3.8	0.9	0.004	−0.09
Well-being (score)	3.6	0.9	3.5	0.9	3.3	1.0	0.008	−0.08

SD: standard deviation; BMI: body mass index; SJL: social jetlag; Mets: metabolic equivalents; AIS: Athens Insomnia Scale; MSFsc: sleep-corrected midpoint of sleep in free days. *p* value was calculated using a Kruskal–Wallis test. *p* < 0.001 was considered as significant and shown in bold. Spearman’s correlation coefficient with more than 0.2 or lower than −0.2 was shown in bold.

**Table 4:** Basic characteristics of each SJL group among women.

	Small SJL		Medium SJL		Large SJL			
	(SJL < 1) N = 2002		(1 ≤ SJL < 2) N = 1138		(2 ≤ SJL) N = 287		Kruskal–Wallis Test	Spearman’s
	Mean	SD	Mean	SD	Mean	SD	*p* Value	Correlation Coefficient
Age (year)	41.8	10.6	40.3	10.0	36.8	10.2	**<0.001**	−0.13
Height (cm)	158.6	5.6	158.7	5.7	158.9	5.4	0.724	0.01
BMI	21.0	7.2	21.1	7.2	21.1	7.7	0.084	0.04
Weight (kg)	57.8	11.3	58.3	10.7	59.3	10.8	0.019	0.05
Daily intake (kcal)	1641.9	285.5	1654.4	275.3	1723.2	287.6	**<0.001**	0.05
Breakfast intake (kcal)	355.2	125.4	342.1	120.5	336.3	134.1	0.002	−0.06
Lunch intake (kcal)	515.9	120.1	519.1	116.4	535.7	130.1	0.092	0.03
Dinner intake (kcal)	560.5	163.6	581.2	159.2	619.0	173.0	**<0.001**	0.09
Breakfast time (hh:mm)	7.9	1.5	7.8	1.3	7.7	1.4	0.193	−0.03
Lunch time (hh:mm)	12.5	1.4	12.5	1.2	12.6	0.9	0.69	−0.01
Dinner time (hh:mm)	19.0	1.7	19.2	1.4	19.3	1.6	**<0.001**	0.10
SD of breakfast time (min)	20.3	34.3	26.8	40.0	35.5	45.8	**<0.001**	0.09
SD of lunch time (min)	18.2	30.8	19.4	35.7	22.5	33.7	0.159	0.02
SD of dinner time (min)	17.2	38.1	18.7	42.4	25.7	51.3	0.032	0.03
Regularities in the rhythm of life (score)	3.8	1.0	3.7	1.0	3.3	1.1	**<0.001**	−0.15
MSFsc (h)	3.5	1.3	3.9	1.1	4.6	0.9	**<0.001**	**0.28**
SJL (h)	0.4	0.3	1.3	0.3	2.3	0.4	**<0.001**	**0.88**
Frequency of breakfast (days/week)	6.1	1.9	6.0	1.8	5.4	2.1	**<0.001**	−0.14
Frequency of late-night snack (days/week)	1.9	2.7	2.0	2.7	1.9	2.5	0.031	0.05
METs (total physical activity)	29.7	37.9	28.0	34.0	31.6	57.8	0.547	−0.02
AIS score	4.3	3.3	4.5	3.4	5.2	3.6	**<0.001**	0.07
Physical strength (score)	2.9	1.0	2.7	0.9	2.6	0.9	**<0.001**	−0.09
Health (score)	3.7	0.9	3.6	0.9	3.5	0.9	**<0.001**	−0.06
Well-being (score)	3.6	1.0	3.5	0.9	3.4	0.9	**<0.001**	−0.06

SD, standard deviation; BMI, body mass index; SJL, social jetlag; METs, metabolic equivalents; AIS, Athens Insomnia Scale; MSFsc, sleep-corrected midpoint of sleep on free days. *p*-values were calculated using the Kruskal–Wallis test. *p* < 0.001 was considered significant and is presented in bold font. A Spearman’s correlation coefficient of more than 0.2 or lower than −0.2 is presented in bold.

**Table 5:** Basic characteristics of each SJL group among men.

	Small SJL		Intermediate SJL		Large SJL			
	(SJL < 1)		(1 ≤ SJL < 2)		(2 ≤ SJL)			
	N = 780		N = 348		N = 71		Kruskal–Wallis Test	Speaman’s
	Mean	SD	Mean	SD	Mean	SD	*p* Value	Correlation Coefficient
Age (year old)	45.9	9.2	45.6	9.1	41.9	10.5	0.007	−0.06
Height (cm)	171.4	5.8	171.5	6.2	171.4	5.5	1.000	0.00
BMI	22.8	7.5	22.8	8.4	25.1	6.1	0.016	0.06
Weight (kg)	73.5	13.4	74.8	14.0	77.2	14.9	0.062	0.06
Daily intake (kcal)	2077.5	394.4	2040.2	431.5	2114.3	433.0	0.541	−0.01
Breakfast intake (kcal)	437.3	153.4	413.9	153.3	413.9	160.6	0.037	−0.07
Lunch intake (kcal)	647.0	145.0	650.8	146.8	630.9	145.9	0.348	−0.01
Dinner intake (kcal)	799.7	196.7	804.5	207.4	820.8	207.0	0.684	0.02
Breakfast time (hh:mm)	7:32	1:16	7:32	1:26	7:29	1:22	0.553	0.03
Lunch time (hh:mm)	12:24	0:54	12:25	1:02	12:34	1:27	0.243	0.05
Dinner time (hh:mm)	19:21	1:25	19:26	1:42	19:35	1:17	0.105	0.06
SD of breakfast time (min)	23.0	38.6	35.5	46.1	51.0	54.4	**<0.001**	0.17
SD of lunch time (min)	17.6	34.0	22.9	35.1	27.8	38.8	**<0.001**	0.11
SD of dinner time (min)	24.9	53.7	36.6	76.0	30.4	58.1	0.030	0.08
Regularities in the rhythm of life (score)	4.0	0.9	3.8	0.9	3.6	1.0	**<0.001**	−0.17
MSFsc (h)	3.1	1.2	3.7	1.2	4.5	1.1	**<0.001**	**0.32**
SJL (h)	0.3	0.3	1.3	0.3	2.4	0.5	**<0.001**	**0.85**
Frequency of breakfast (days/week)	6.3	1.8	6.1	1.9	5.8	2.2	**<0.001**	−0.11
Frequency of late night snack (days/week)	2.1	2.9	2.3	2.9	2.7	2.8	0.071	0.06
Mets (total physical activity)	37.7	38.8	33.1	36.4	45.9	53.2	0.004	−0.06
AIS score	3.8	3.0	4.5	3.6	4.6	3.8	0.005	0.09
Physical strength (score)	3.1	0.9	3.0	0.9	2.9	1.0	0.011	−0.09
Health (score)	3.9	0.8	3.6	0.9	3.8	0.8	**<0.001**	−0.15
Well-being (score)	3.6	0.9	3.4	1.0	3.5	0.9	**<0.001**	−0.10

SD: standard deviation; BMI: body mass index; SJL: social jetlag; Mets: metabolic equivalents; AIS: Athens Insomnia Scale; MSFsc: sleep-corrected midpoint of sleep in free days. *p* value was calculated using a Kruskal–Wallis test. *p* < 0.001 was considered as significant and shown in bold. Spearman’s correlation coefficient with more than 0.2 or lower than −0.2 was shown in bold.

**Table 6:** Association between chronotype/SJL and daily total nutrient intake by multiple regression analyses.

Dependent Variable	Independent Variable: Chronotype					Independent Variable: SJL				
Daily Total Intake	R^2^	B	Min	Max	*p* Value	R^2^	B	Min	Max	*p* Value
Energy	0.25	5.19	−2.40	12.79	0.180	0.25	22.08	8.45	35.71	0.002
Protein	0.30	−0.11	−0.47	0.24	0.523	0.30	−0.85	−1.48	−0.22	0.008
Lipid	0.54	0.29	0.05	0.54	0.021	0.54	0.46	0.01	0.90	0.047
Carbohydrate	0.59	0.21	−0.57	1.00	0.592	0.59	0.65	−0.76	2.06	0.365
Sodium	0.39	−9.48	−26.48	7.53	0.275	0.39	32.33	1.79	62.88	0.038
Potassium	0.14	−40.24	−53.90	−26.59	**<0.001**	0.13	−44.13	−68.72	−19.54	**<0.001**
Cholesterol	0.09	0.42	−1.21	2.05	0.613	0.09	−1.88	−4.81	1.04	0.207
Fiber	0.08	−0.49	−0.62	−0.36	**<0.001**	0.08	−0.74	−0.97	−0.51	**<0.001**
Saturated fatty acid	0.39	0.10	0.01	0.19	0.025	0.39	0.18	0.02	0.34	0.025
Alcohol	0.09	−0.29	−0.48	−0.10	0.002	0.09	0.16	−0.17	0.49	0.348
Calcium	0.05	−0.76	−6.43	4.91	0.793	0.05	−14.15	−24.33	−3.96	0.006
Magnesium	0.10	−3.63	−5.82	−1.44	**<0.001**	0.10	−7.41	−11.34	−3.48	**<0.001**
Phosphorus	0.31	−14.26	−18.98	−9.54	**<0.001**	0.31	−13.87	−22.37	−5.37	**<0.001**
Iron	0.01	0.03	−0.11	0.17	0.663	0.01	−0.06	−0.31	0.19	0.630
Zinc	0.10	0.05	−0.07	0.16	0.426	0.10	−0.06	−0.26	0.14	0.570
Vitamin A	0.02	9.40	−5.29	24.09	0.210	0.02	−6.28	−32.68	20.13	0.641
Vitamin D	0.00	0.18	−0.64	0.99	0.670	0.00	1.31	−0.16	2.77	0.080
Vitamin E	0.00	0.54	0.01	1.07	0.047	0.00	−0.05	−1.01	0.91	0.915
Vitamin K	0.04	−12.63	−15.65	−9.61	**<0.001**	0.03	−17.14	−22.59	−11.70	**<0.001**
Vitamin B1	0.01	0.20	0.03	0.36	0.018	0.00	−0.14	−0.43	0.15	0.349
Vitamin B2	0.00	0.09	−0.08	0.26	0.291	0.00	−0.16	−0.46	0.14	0.297
Vitamin B3	0.03	0.10	−0.42	0.62	0.701	0.03	−0.31	−1.24	0.63	0.521
Vitamin B6	0.00	0.02	−0.17	0.21	0.810	0.00	−0.09	−0.43	0.26	0.627
Vitamin B12	0.00	−0.07	−0.99	0.85	0.880	0.00	−1.05	−2.71	0.60	0.213
Folate	0.02	−3.41	−8.11	1.29	0.155	0.02	−8.99	−17.43	−0.55	0.037
Vitamin B5	0.02	−0.06	−0.21	0.09	0.427	0.02	−0.34	−0.61	−0.07	0.014
Vitamin C	0.00	8.35	0.05	16.64	0.049	0.00	11.55	−3.36	26.45	0.129

Multiple regression analyses were conducted with each nutrient intake amount as a dependent variable and chronotype (1: morning, 2: intermediate, and 3: evening) or SJL (1: small SJL, 2: medium SJL, and 3: large SJL) as an independent variable in each calculation. Age, gender, BMI, and total daily intake were used as confounding factors. Significant *p*-values of the independent variable (chronotype or SJL) are presented in bold (*p* < 0.001).

**Table 7:** Association between chronotype /SJL and breakfast nutrient intake by multiple regression analyses.

Dependent Variable	Independent Variable: Chronotype					Independent Variable: SJL				
Brakfast Intake	R^2^	B	Min	Max	*p* Value	R^2^	B	Min	Max	*p* Value
Energy	0.29	−21.88	−24.66	−19.09	**<0.001**	0.26	−14.82	−19.89	−9.75	**<0.001**
Protein	0.12	−1.04	−1.21	−0.87	**<0.001**	0.10	0.01	0.00	0.01	**<0.001**
Lipid	0.19	−0.71	−0.83	−0.59	**<0.001**	0.17	−0.51	−0.73	−0.30	**<0.001**
Carbohydrate	0.23	−2.99	−3.41	−2.56	**<0.001**	0.20	−1.71	−2.48	−0.94	**<0.001**
Sodium	0.14	−50.84	−59.16	−42.52	**<0.001**	0.11	−24.07	−39.16	−8.97	0.002
Potassium	0.08	−44.69	−51.56	−37.81	**<0.001**	0.05	−39.94	−52.36	−27.52	**<0.001**
Cholesterol	0.06	−6.71	−7.83	−5.59	**<0.001**	0.03	−4.87	−6.90	−2.83	**<0.001**
Fiber	0.04	−0.41	−0.48	−0.34	**<0.001**	0.02	−0.42	−0.55	−0.30	**<0.001**
Saturated fatty acid	0.15	−0.18	−0.22	−0.14	**<0.001**	0.14	−0.13	−0.20	−0.06	**<0.001**
Alcohol	0.05	−0.02	−0.02	−0.01	**<0.001**	0.04	−0.01	−0.03	0.00	0.058
Calcium	0.03	−8.87	−11.96	−5.79	**<0.001**	0.03	−6.70	−12.24	−1.16	00.018
Magnesium	0.05	−4.74	−5.85	−3.63	**<0.001**	0.04	−4.53	−6.53	−2.53	**<0.001**
Phosphorus	0.13	−19.15	−21.56	−16.75	**<0.001**	0.09	−14.67	−19.07	−10.26	**<0.001**
Iron	0.00	−0.12	−0.23	−0.02	0.023	0.00	−0.03	−0.22	0.16	0.752
Zinc	0.04	−0.10	−0.16	−0.05	**<0.001**	0.03	−0.06	−0.15	0.04	0.225
Vitamin A	0.02	−6.93	−13.17	−0.70	0.029	0.02	0.03	−11.15	11.21	0.996
Vitamin D	0.00	−0.42	−0.72	−0.11	0.008	0.00	−0.24	−0.80	0.31	0.388
Vitamin E	0.00	−0.24	−0.57	0.09	0.149	0.00	0.16	−0.43	0.74	0.594
Vitamin K	0.04	−9.77	−11.46	−8.09	**<0.001**	0.02	−9.57	−12.63	−6.52	**<0.001**
Vitamin B1	0.00	−0.04	−0.13	0.06	0.422	0.00	−0.08	−0.25	0.09	0.348
Vitamin B2	0.00	−0.07	−0.18	0.03	0.160	0.00	−0.09	−0.28	0.09	0.318
Vitamin B3	0.01	−0.40	−0.71	−0.10	0.010	0.01	−0.24	−0.78	0.31	0.397
Vitamin B6	0.00	−0.08	−0.18	0.03	0.151	0.00	−0.14	−0.33	0.05	0.137
Vitamin B12	0.00	−0.07	−0.45	0.31	0.722	0.00	−0.14	−0.83	0.54	0.681
Folate	0.01	−5.75	−8.60	−2.90	**<0.001**	0.01	−4.72	−9.83	0.39	0.070
Vitamin B5	0.01	−0.18	−0.27	−0.08	**<0.001**	0.01	−0.22	−0.39	−0.05	0.013
Vitamin C	0.00	2.82	−1.80	7.43	0.232	0.00	2.10	−6.18	10.37	0.620

Multiple regression analyses were conducted with each nutrient intake amount as a dependent variable and chronotype (1: morning, 2: intermediate, and 3: evening) or SJL (1: small SJL, 2: medium SJL, and 3: large SJL) as an independent variable in each calculation. Age, gender, BMI, and total daily intake were used as confounding factors. Significant *p*-values of the independent variable (chronotype or SJL) are presented in bold (*p* < 0.001).

**Table 8:** Association between chronotype /SJL and lunch nutrient intake by multiple regression analyses.

Dependent Variable	Independent Variable: Chronotype					Independent Variable: SJL				
Lunch Intake	R^2^	B	Min	Max	*p* Value	R^2^	B	Min	Max	*p* Value
Energy	0.46	−2.67	−5.12	−0.22	0.033	0.46	−0.91	−5.31	3.50	0.686
Protein	0.18	−0.21	−0.36	−0.07	0.004	0.18	−0.27	−0.53	−0.01	0.040
Lipid	0.26	0.06	−0.07	0.19	0.355	0.26	0.03	−0.20	0.26	0.808
Carbohydrate	0.38	−0.48	−0.85	−0.11	0.010	0.38	−0.09	−0.75	0.57	0.791
Sodium	0.23	−6.71	−15.63	2.21	0.140	0.23	10.58	−5.44	26.60	0.196
Potassium	0.05	−15.47	−20.92	−10.02	**<0.001**	0.04	−17.32	−27.10	−7.53	**<0.001**
Cholesterol	0.03	−2.02	−2.99	−1.06	**<0.001**	0.03	−4.26	−5.99	−2.53	**<0.001**
Fiber	0.03	−0.15	−0.20	−0.10	**<0.001**	0.03	−0.23	−0.32	−0.14	**<0.001**
Saturated fatty acid	0.18	0.02	−0.02	0.06	0.280	0.18	−0.01	−0.07	0.06	0.895
Alcohol	0.04	−0.03	−0.05	−0.01	0.004	0.03	0.00	−0.04	0.04	0.883
Calcium	0.02	−0.35	−1.96	1.27	0.673	0.02	−4.08	−6.97	−1.18	0.006
Magnesium	0.05	−1.48	−2.14	−0.81	**<0.001**	0.05	−2.19	−3.38	−1.00	**<0.001**
Phosphorus	0.13	−5.64	−7.70	−3.57	**<0.001**	0.12	−5.60	−9.32	−1.89	0.003
Iron	0.03	−0.03	−0.05	0.00	0.057	0.03	−0.06	−0.10	−0.01	0.015
Zinc	0.05	−0.03	−0.06	0.01	0.136	0.05	0.02	−0.04	0.08	0.576
Vitamin A	0.01	−8.26	−12.76	−3.76	**<0.001**	0.00	−6.00	−14.10	2.09	0.146
Vitamin D	0.00	0.22	−0.16	0.60	0.257	0.00	0.82	0.15	1.50	0.017
Vitamin E	0.01	0.03	−0.06	0.11	0.551	0.01	−0.07	−0.22	0.08	0.375
Vitamin K	0.01	−2.87	−4.02	−1.72	**<0.001**	0.01	−5.45	−7.52	−3.39	**<0.001**
Vitamin B1	0.00	0.02	−0.01	0.04	0.177	0.00	0.01	−0.04	0.05	0.711
Vitamin B2	0.00	0.02	−0.01	0.05	0.206	0.00	0.00	−0.06	0.06	0.993
Vitamin B3	0.09	−0.11	−0.18	−0.03	0.005	0.08	−0.09	−0.22	0.04	0.191
Vitamin B6	0.00	0.01	−0.02	0.04	0.499	0.00	0.00	−0.05	0.05	0.991
Vitamin B12	0.00	−0.10	−0.25	0.05	0.205	0.00	−0.16	−0.43	0.11	0.247
Folate	0.01	−2.78	−4.14	−1.41	**<0.001**	0.01	−3.83	−6.27	−1.38	0.002
Vitamin B5	0.03	−0.02	−0.05	0.00	0.085	0.03	−0.05	−0.10	0.00	0.048
Vitamin C	0.00	0.33	−1.18	1.84	0.671	0.00	−1.39	−4.11	1.32	0.314

Multiple regression analyses were conducted with each nutrient intake amount as a dependent variable and chronotype (1: morning, 2: intermediate, and 3: evening) or SJL (1: small SJL, 2: medium SJL, and 3: large SJL) as an independent variable in each calculation. Age, gender, body mass index, and total daily intake were used as confounding factors. Significant *p*-values of the independent variable (chronotype or SJL) are presented in bold (*p* < 0.001).

**Table 9:** Association between chronotype/SJL and dinner nutrient intake by multiple regression analyses.

Dependent Variable	Independent Variable: Chronotype					Independent Variable: SJL				
Dinner Intake	R^2^	B	Min	Max	*p* Value	R^2^	B	Min	Max	*p* Value
Energy	0.55	15.76	12.55	18.98	**<0.001**	0.55	16.54	10.72	22.36	**<0.001**
Protein	0.29	0.70	0.52	0.87	**<0.001**	0.28	0.64	0.33	0.96	**<0.001**
Lipid	0.33	0.63	0.47	0.79	**<0.001**	0.32	0.72	0.43	1.00	**<0.001**
Carbohydrate	0.37	2.67	2.21	3.12	**<0.001**	0.35	2.12	1.28	2.95	**<0.001**
Sodium	0.23	41.60	31.22	51.99	**<0.001**	0.22	43.06	24.33	61.78	**<0.001**
Potassium	0.12	9.24	2.46	16.01	0.008	0.12	−0.64	−12.79	11.52	0.918
Cholesterol	0.11	1.90	0.90	2.90	**<0.001**	0.11	2.20	0.41	3.99	0.016
Fiber	0.09	0.06	0.00	0.11	0.039	0.09	−0.06	−0.16	0.04	0.209
Saturated fatty acid	0.27	0.17	0.12	0.22	**<0.001**	0.27	0.21	0.12	0.30	**<0.001**
Alcohol	0.09	−0.34	−0.51	−0.17	**<0.001**	0.09	0.16	−0.14	0.47	0.294
Calcium	0.03	0.25	−1.94	2.45	0.821	0.03	−1.53	−5.47	2.41	0.447
Magnesium	0.09	0.61	−0.35	1.57	0.210	0.09	−0.35	−2.07	1.38	0.694
Phosphorus	0.22	6.63	4.06	9.20	**<0.001**	0.22	6.42	1.80	11.04	0.006
Iron	0.02	0.08	0.02	0.13	0.009	0.02	0.09	−0.01	0.19	0.092
Zinc	0.05	0.09	0.02	0.16	0.009	0.05	−0.03	−0.15	0.09	0.572
Vitamin A	0.01	15.06	3.99	26.14	0.008	0.00	1.88	−18.04	21.79	0.854
Vitamin D	0.00	0.08	−0.14	0.31	0.468	0.00	0.16	−0.25	0.57	0.444
Vitamin E	0.00	0.26	0.05	0.46	0.014	0.00	−0.10	−0.47	0.27	0.607
Vitamin K	0.02	−0.35	−2.05	1.35	0.688	0.02	−1.90	−4.95	1.16	0.223
Vitamin B1	0.00	0.08	0.02	0.14	0.010	0.00	−0.04	−0.15	0.07	0.462
Vitamin B2	0.00	0.04	−0.03	0.11	0.217	0.00	−0.08	−0.21	0.04	0.205
Vitamin B3	0.01	0.43	0.14	0.71	0.004	0.01	0.18	−0.34	0.70	0.487
Vitamin B6	0.00	0.03	−0.03	0.09	0.353	0.00	−0.03	−0.15	0.08	0.556
Vitamin B12	0.00	0.17	−0.54	0.88	0.644	0.00	−0.40	−1.67	0.88	0.543
Folate	0.02	1.69	−0.16	3.54	0.073	0.02	0.21	−3.11	3.53	0.900
Vitamin B5	0.01	0.05	−0.01	0.10	0.093	0.01	−0.02	−0.12	0.08	0.741
Vitamin C	0.00	1.49	−1.97	4.94	0.399	0.00	5.24	−0.96	11.44	0.098

Multiple regression analyses were conducted with each nutrient intake amount as a dependent variable and chronotype (1: morning, 2: intermediate, and 3: evening) or SJL (1, small SJL, 2: medium SJL, and 3: large SJL) as an independent variable in each calculation. Age, gender, body mass index, and total daily intake were used as confounding factors. Significant *p*-values of the independent variable (chronotype or SJL) are presented in bold (*p* < 0.001).

## Data Availability

The data used in this study are the property of the company and will not be released to the public. However, the data will be provided to researchers, upon request, for research purposes.

## Supplementary Materials

The following supporting information can be downloaded at https://www.mdpi.com/article/10.3390/nu15092165/s1, Table S1: Association between chronotype/SJL and % nutrient intake in the breakfast/total daily intake by multiple regression analyses, Table S2: Association between chronotype/SJL and % nutrient intake in dinner/total daily intake by multiple regression analyses, Table S3: Association between BMI and chronotype or SJL.
