# Supplementary material for: Association of Eating Pattern, Chronotype, and Social Jetlag: A Cross-Sectional Study Using Data Accumulated in a Japanese Food-Logging Mobile Health Application
Source: Nutrients. 2023 Apr 30;15(9):2165. doi: 10.3390/nu15092165 (PMC10181260; doi:10.3390/nu15092165)
Supplement: Supplementary file 1 [file nutrients-15-02165-s001.zip › nutrients-2299757-supplementary.pdf]

Table S1. Association between chronotype/SJL and % nutrient intake in the breakfast/total daily intake by multiple regression analyses.

Dependent variable	Independent variable: chronotype					Independent variable: SJL				
% ratio in breakfast	**R2**	**B**	**Min**	**Max**	**P value**	**R2**	**B**	**Min**	**Max**	**P value**
Protein	0.036	-2.416	-2.783	-2.048	<.001	0.009	-1.19	-1.567	-0.812	<.001
Lipid	0.031	-2.156	-2.518	-1.794	<.001	0.008	-0.941	-1.312	-0.57	<.001
Carbohydrate	0.045	-2.547	-2.899	-2.195	<.001	0.008	-0.88	-1.243	-0.516	<.001
Sodium	0.031	-2.415	-2.829	-2.001	<.001	0.008	-0.904	-1.328	-0.481	<.001
Potassium	0.039	-2.45	-2.871	-2.029	<.001	0.017	-1.196	-1.625	-0.767	<.001
Cholesterol	0.003	-4.405	-10.659	1.85	0.167	0.003	-0.685	-7.012	5.641	0.832
Fiber	0.023	-2.559	-3.042	-2.075	<.001	0.005	-1.156	-1.651	-0.661	<.001
Saturated fatty acid	0.03	-2.093	-2.505	-1.682	<.001	0.014	-0.948	-1.369	-0.527	<.001
Alcohol	0.005	-1.222	-1.809	-0.635	<.001	0.003	-0.758	-1.353	-0.163	0.013
Calcium	0.028	-2.256	-2.821	-1.692	<.001	0.017	-0.838	-1.414	-0.262	0.004
Magnesium	0.029	-2.287	-2.739	-1.835	<.001	0.012	-0.999	-1.461	-0.536	<.001
Phosphorus	0.051	-2.69	-3.068	-2.311	<.001	0.018	-1.175	-1.565	-0.785	<.001
Iron	0.014	-2.478	-3.103	-1.852	<.001	0.001	-0.904	-1.543	-0.266	0.006
Zinc	0.018	-2.305	-2.839	-1.771	<.001	0.004	-0.729	-1.274	-0.183	0.009
Vitamin A	0.017	-2.252	-2.996	-1.508	<.001	0.01	-0.614	-1.371	0.144	0.112
Vitamin D	0.013	-2.551	-3.374	-1.729	<.001	0.006	-0.628	-1.465	0.209	0.142
Vitamin E	0.021	-2.941	-3.701	-2.182	<.001	0.01	-0.968	-1.742	-0.194	0.014
Vitamin K	0.031	-4.059	-4.795	-3.322	<.001	0.01	-1.752	-2.506	-0.997	<.001
Vitamin B1	0.009	-2.526	-3.435	-1.616	<.001	0.003	-0.628	-1.553	0.297	0.184
Vitamin B2	0.013	-2.711	-3.576	-1.847	<.001	0.006	-0.983	-1.863	-0.103	0.029
Vitamin B3	0.02	-2.468	-3.05	-1.886	<.001	0.008	-0.966	-1.56	-0.372	0.001
Vitamin B6	0.007	-2.057	-2.928	-1.186	<.001	0.003	-0.826	-1.711	0.059	0.067
Vitamin B12	0.005	-1.728	-2.461	-0.996	<.001	0.001	-0.514	-1.258	0.231	0.176
Folate	0.019	-2.429	-3.04	-1.818	<.001	0.008	-0.88	-1.503	-0.257	0.006
Vitamin B5	0.019	-2.655	-3.335	-1.975	<.001	0.009	-1.12	-1.814	-0.427	0.002
Vitamin C	0.008	-1.837	-2.747	-0.928	<.001	0.005	-0.724	-1.647	0.2	0.125

Multiple regression analyses were conducted with each nutrient intake amount (% breakfast/daily total) as a dependent variable and chronotype (1: morning, 2: intermediate, and 3: evening) or SJL (1: small SJL, 2: medium SJL, and 3: large SJL) as an independent variable in each calculation. Age, gender, BMI, and total daily intake were sed as confounding factors. Significant P-values of the independent variable (chronotype or SJL) are presented in bold ($P < 0.001$).

Table S2. Association between chronotype/SJL and % nutrient intake in dinner/total daily intake by multiple regression analyses.

Dependent variable	Independent variable: chronotype					Independent variable: SJL				
% ratio in dinner	**R2**	**B**	**Min**	**Max**	**P value**	**R2**	**B**	**Min**	**Max**	**P value**
Protein	0.036	1.806	1.406	2.206	<.001	0.026	1.118	0.71	1.526	<.001
Lipid	0.036	1.51	1.084	1.936	<.001	0.028	0.668	0.234	1.103	0.003
Carbohydrate	0.058	2.395	2.039	2.751	<.001	0.027	0.814	0.447	1.182	<.001
Sodium	0.026	2.28	1.838	2.723	<.001	0.007	0.82	0.367	1.273	<.001
Potassium	0.024	1.738	1.313	2.164	<.001	0.012	0.74	0.307	1.173	<.001
Cholesterol	0.025	1.498	0.784	2.213	<.001	0.025	1.435	0.711	2.16	<.001
Fiber	0.028	2.138	1.702	2.573	<.001	0.012	0.954	0.51	1.397	<.001
Saturated fatty acid	0.045	1.477	1.016	1.938	<.001	0.038	0.682	0.212	1.153	0.005
Alcohol	0.017	0.251	-0.905	1.408	0.67	0.018	1.099	-0.077	2.274	0.067
Calcium	0.005	0.534	0.058	1.01	0.028	0.005	0.55	0.067	1.033	0.026
Magnesium	0.012	1.463	1.02	1.905	<.001	0.006	0.818	0.367	1.268	<.001
Phosphorus	0.037	2.078	1.688	2.468	<.001	0.019	1.008	0.608	1.408	<.001
Iron	0.016	1.597	1.046	2.147	<.001	0.012	0.991	0.431	1.551	<.001
Zinc	0.015	2.037	1.501	2.573	<.001	0.004	0.536	-0.011	1.083	0.055
Vitamin A	0.007	2.005	1.26	2.75	<.001	0.003	1.14	0.382	1.898	0.003
Vitamin D	0.006	1.448	0.625	2.272	<.001	0.004	0.72	-0.117	1.556	0.092
Vitamin E	0.005	1.628	1.009	2.246	<.001	0.001	0.852	0.223	1.481	0.008
Vitamin K	0.02	3.292	2.563	4.022	<.001	0.008	1.765	1.019	2.51	<.001
Vitamin B1	0.006	1.108	0.341	1.875	0.005	0.005	0.681	-0.098	1.46	0.087
Vitamin B2	0.002	0.905	0.202	1.608	0.012	0.002	0.672	-0.042	1.385	0.065
Vitamin B3	0.01	1.803	1.247	2.358	<.001	0.004	1.006	0.44	1.571	<.001
Vitamin B6	0.008	0.751	-0.009	1.512	0.053	0.008	0.795	0.023	1.568	0.043
Vitamin B12	0.013	1.933	1.132	2.734	<.001	0.009	0.911	0.096	1.726	0.028
Folate	0.009	1.691	1.108	2.273	<.001	0.004	0.848	0.255	1.442	0.005
Vitamin B5	0.005	1.394	0.825	1.964	<.001	0.002	0.922	0.344	1.501	0.002
Vitamin C	0.001	0.329	-0.453	1.111	0.41	0.001	0.497	-0.297	1.291	0.22

Multiple regression analyses were conducted with each nutrient intake amount (% dinner/daily total) as a dependent variable and chronotype (1: morning, 2: intermediate, and 3: evening) or SJL (1: small SJL, 2: medium SJL, and 3: large SJL) as an independent variable in each calculation. Age, gender, BMI, and total daily intake were sed as confounding factors. Significant P-values of the independent variable (chronotype or SJL) are presented in bold (P < 0.001).

Table S3. Association between BMI and chronotype or SJL.

Dependent variable	Adjusted R squared	*p* value	Explanatory variable	Standardization coefficient β	*p* value	95% CI for B	
						minimum	maximum
			Gender	-0.107	<.001	-2.317	-1.324
BMI	0.017	<.001	Age	0.057	<.001	0.019	0.062
			Chronotype	0.057	<.001	0.304	0.969
			Gender	-0.104	<.001	-2.268	-1.276
BMI	0.014	<.001	Age	0.046	0.002	0.012	0.054
			SJL	0.026	0.076	-0.032	0.644

Multiple regression analyses were conducted with BMI as a dependent variable and chronotype (1: morning, 2: intermediate, and 3: evening) or SJL (1: small SJL, 2: medium SJL, and 3: large SJL) as an independent variable in each calculation. Age, gender, BMI, and total daily intake were sed as confounding factors.
